# Mapping annual 10-m soybean cropland with spatiotemporal sample migration

**DOI:** 10.1038/s41597-024-03273-5

**Published:** 2024-05-02

**Authors:** Hongchi Zhang, Zihang Lou, Dailiang Peng, Bing Zhang, Wang Luo, Jianxi Huang, Xiaoyang Zhang, Le Yu, Fumin Wang, Linsheng Huang, Guohua Liu, Shuang Gao, Jinkang Hu, Songlin Yang, Enhui Cheng

**Affiliations:** 1 Key Laboratory of Digital Earth Science, Aerospace Information Research Institute, Chinese Academy of Sciences, Beijing, 100094 China; 2 International Research Center of Big Data for Sustainable Development Goals, Beijing, 100094 China; 3 University of Chinese Academy of Sciences, Beijing, 100094 China; 4 Jiangxi Nuclearindustry Surveying and Mapping Institute Group Co., Ltd, Nanchang, 330038 China; 5 College of Land Science and Technology, China Agricultural University, Beijing, 100083 China; 6 Geospatial Sciences Center of Excellence, Department of Geography Geospatial Sciences, South Dakota State University, Brookings, SD 57007 USA; 7 Department of Earth System Science, Tsinghua University, Beijing, 100084 China; 8 Institute of Applied Remote Sensing & Information Technology, Zhejiang University, Hangzhou, 310058 China; 9 National Engineering Research Center for Agro-Ecological Big Data Analysis & Application, Anhui University, Hefei, 230601 China; 10 Innovation Academy for Microsatellites, Chinese Academy of Sciences, Shanghai, 200120 China

**Keywords:** Environmental sciences, Ecology

## Abstract

China, as the world’s biggest soybean importer and fourth-largest producer, needs accurate mapping of its planting areas for global food supply stability. The challenge lies in gathering and collating ground survey data for different crops. We proposed a spatiotemporal migration method leveraging vegetation indices’ temporal characteristics. This method uses a feature space of six integrals from the crops’ phenological curves and a concavity-convexity index to distinguish soybean and non-soybean samples in cropland. Using a limited number of actual samples and our method, we extracted features from optical time-series images throughout the soybean growing season. The cloud and rain-affected data were supplemented with SAR data. We then used the random forest algorithm for classification. Consequently, we developed the 10-meter resolution ChinaSoybean10 maps for the ten primary soybean-producing provinces from 2019 to 2022. The map showed an overall accuracy of about 93%, aligning significantly with the statistical yearbook data, confirming its reliability. This research aids soybean growth monitoring, yield estimation, strategy development, resource management, and food scarcity mitigation, and promotes sustainable agriculture.

## Background & Summary

Soybeans (*Glycine max*) are extensively grown for their high oil content, abundant protein, and substantial contribution to energy production^1^. Over the last two decades, soybeans have consistently played a vital role in the Chinese diet^1^ and have been a crucial source of oil and animal feed. China is the world’s largest consumer of soybeans^2^. China produced 20.28 million tons of soybeans in 2022 while importing an additional 91.08 million tons from countries such as Brazil, the United States, and Argentina^3^. Forecasts indicate that China’s soybean demand will reach around 133 million tons by 2035, increasing the pressure on domestic production and imports^4,5^. Utilizing satellite-based earth observation data for national-scale mapping of soybean is a cost-effective method to gather comprehensive information^6,7^. This spatial information can effectively reveal soybeans distribution, laying a strong foundation for agricultural management and yield prediction^6,7^.

Since the late 1990s, remote sensing imagery has progressively assumed a pivotal role in the identification and monitoring of crops^8,9^. Numerous researchers have undertaken nationwide and regional-scale crop mapping utilizing remote sensing data^10–13^. In the early years, researchers typically utilized single-phase or multi-temporal images as primary data sources for crop remote sensing identification, obtaining one or more images during the critical growing season to facilitate crop identification^14–16^. This method has a small amount of data and computational complexity, but the crop identification features extracted are relatively limited, so the accuracy is relatively low. Time-series data has garnered increased attention in recent years for crop identification due to its capacity to capture crop growth patterns accurately. Several studies have employed time-series data for precise crop identification^17–21^. Regarding classification, machine learning algorithms, with their robust self-learning and generalization capabilities, have consistently exhibited exceptional accuracy and stability in classifying crops through remote sensing, rendering them among the most widely utilized techniques^22,23^. Despite substantial advancements in data and methods for remote sensing crop classification, accurately distinguishing specific crop types such as soybeans, corn, and wheat from imagery remains a formidable challenging^24^.

While various crops exhibit categorical differences, their shared vegetative characteristics often lead to subtle spectral distinctions. Consequently, it is essential to incorporate vegetation index and specific spectral bands to capture the distinct biophysical attributes of crops, particularly soybeans, which frequently encounter pronounced spectral overlap with certain other crops^8^. Previous studies have highlighted a noteworthy feature of soybeans related to reduced canopy water content during the growing season, distinguishing them from some other crops at comparable phenological stages^25,26^. The short-wave infrared (SWIR) bands effectively capture this information^27,28^. Additionally, the red-edge bands (Sentinel-2) and vegetation index derived from these bands, such as Red Edge Normalized Difference Vegetation Index (RENDVI) and Red Edge Position Index (REPI), play a crucial role in discriminating soybean from corn, thereby enhancing the classifier’s accuracy in soybean classification^20,29^.

Methods for crop recognition relying on spectral or vegetation indexes as input features often depend on specific datasets and ground references. However, obtaining sufficient ground-truth crop data typically constitutes the most demanding, time-consuming, and expensive aspect of crop mapping^30^. Consequently, numerous researchers have focused on studying crop mapping in scenarios with either no samples or limited samples. For instance, researchers have explored the physicochemical characteristics of crops by analysing their spectral and vegetation index profiles. They have developed techniques such as knowledge transfer topologies^31^, multi-temporal Gaussian mixture models^32^, and the integrated Greenness and Water Content Composite Index (GWCCI)^27^ for mapping soybeans and corn in space. However, these methods are often more suitable for regions with extensive soybean or corn cultivation and may be sensitive to other types of vegetation or crops in areas with intricate planting patterns. Consequently, some researchers have chosen to employ crop ground-truth samples from preceding years for feature transfer or sample migration^33,34^. Despite potential variations in certain crop features across time and space, these characteristics tend to exhibit a consistent level of stability^35^. Supervised learning methods are subsequently employed to conduct crop classification in subsequent years^22,33,36^. Results obtained through these limited or zero-sample methods may not be optimal but still demonstrate acceptable performance and accuracy.

Developing nationwide crop maps presents a formidable challenge that necessitates the availability of high-quality remote sensing data, abundant ground-truth crop data, and well-designed classification methods^37^. In China, soybean cultivation spans approximately 8% of arable land, with nearly half concentrated in the northeastern region^3^. However, in the northeast, soybeans cultivation merely encompasses 5% of the available arable land^38^. These factors highlight the distinctive nature of soybean cultivation in China, characterized by a small cultivation area, dispersed plots, and considerable annual variations. Consequently, the creation of nationwide soybean distribution maps is a highly intricate undertaking. Majority of the current spatial maps for soybean in China are primarily concentrated in the northeastern region^24,25,39^, and only one product in 2019 covers the whole country (GLAD maize and soybean map)^13^. One of the primary obstacles in generating high-resolution soybean maps lies in the absence of reliable ground-truth data. While some methods have been devised to classify and map soybeans with minimal or no samples^27,31^, as well as perform early-season classification using data using previous years^22^, these approaches possess limitations, particularly in the diverse crop landscape of Huang-Huai-Hai Plain and the Middle-Lower Yangtze Plain. Moreover, intricate planting practices on small farms are affected by various factors, including economic shifts and alterations in land use policies^40,41^, thereby leading to annual variations in crop types and rendering crop identification more challenging. Despite earnest efforts to map crops at a 10-meter resolution throughout China, soybean mapping remains restricted, especially in regions like Sichuan, Anhui, and Henan, which boast high levels of soybean production and lie beyond the primary Northeastern cultivation areas.

In response to the challenges posed by the lack of high spatial resolution soybean mapping and the absence of ground truth samples in China, our study aims to create China’s annual soybean map based on limited samples and spatiotemporal migration methods. Initially, based on the growth physical and chemical characteristics of soybeans, we generate samples from limited ground survey samples for the target year and region. We then employed random forest classification, utilizing soybean temporal features derived from time series of vegetation indexes and spectral bands as input. These features encompassed statistical measures, phenological characteristics, and harmonic fitting parameters. In regions with frequent cloud cover or a shorter soybean growing season, SAR data were incorporated to complement features. This involved utilizing statistical features and principal component features of backscatter coefficients and their combinations. Our comprehensive approach enables nationwide mapping of soybean planting areas. We successfully generated spatial maps of soybean cultivation for 10 provinces in China, including Heilongjiang, Jilin, Inner Mongolia, Henan, Sichuan, and others, covering the years from 2019 to 2022 at a 10-meter resolution.

## Methods

**Fig. 1:**
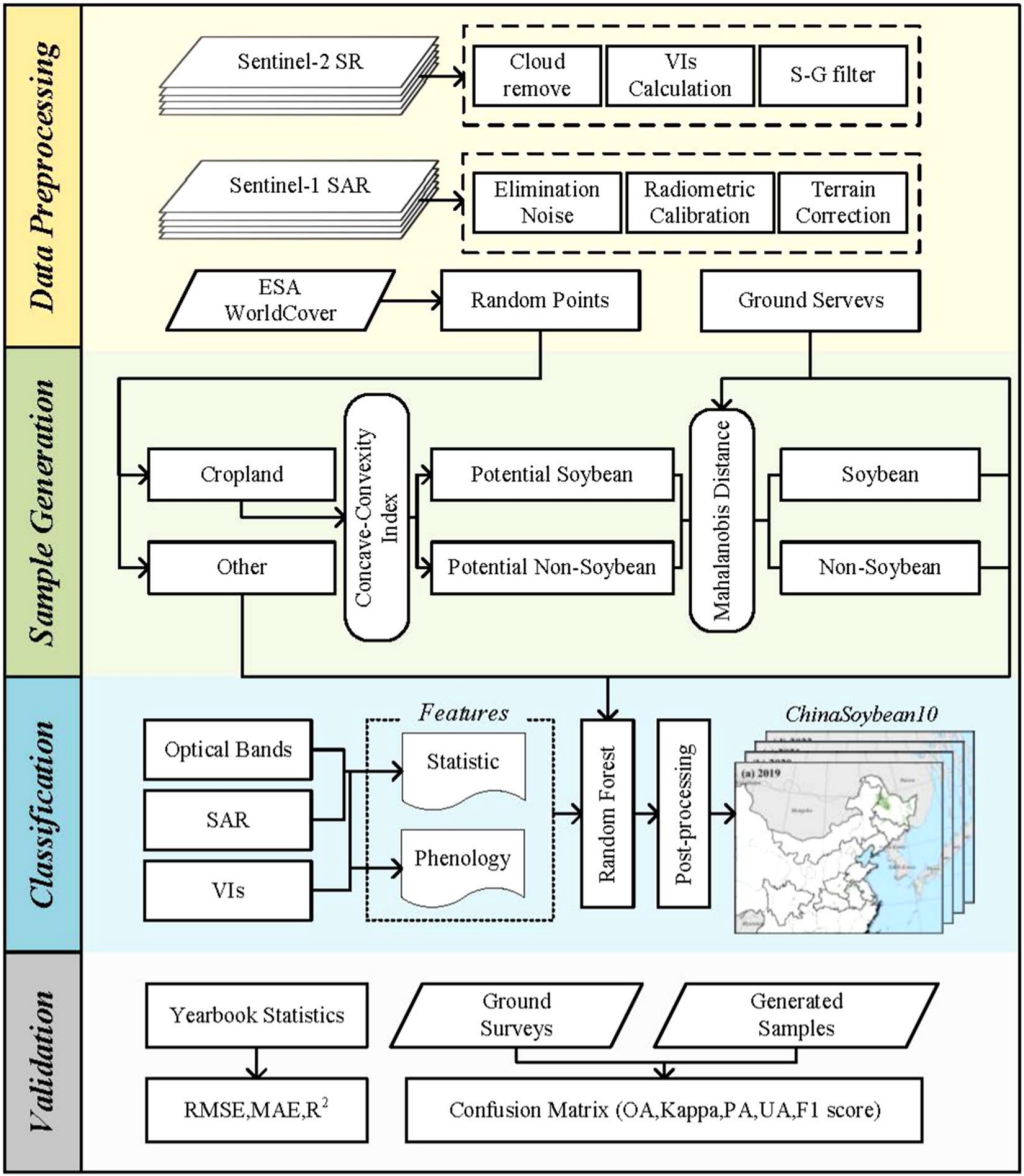
Workflow for mapping soybean planting areas using the sample-generation and pixel-based algorithm. Sentinel-2 SR, sentinel-2 surface reflectance products in Google Earth Engine; Sentinel-1 SAR, a dual-polarization C-band Synthetic Aperture Radar data at 5.405 GHz; S-G filter, Savitzky-Golay filter; ESA, European Space Agency; VIs, Vegetation Indices; RMSE, root-mean-square error; MAE, mean absolute error; R2, R-squared; OA, overall accuracy; PA, producer’s accuracy; UA, user’s accuracy.

Our soybean cropland mapping process includes four steps, as illustrated in Fig. 1: data preprocessing, sample generation, classification and validation.

### Study Area

**Fig. 2:**
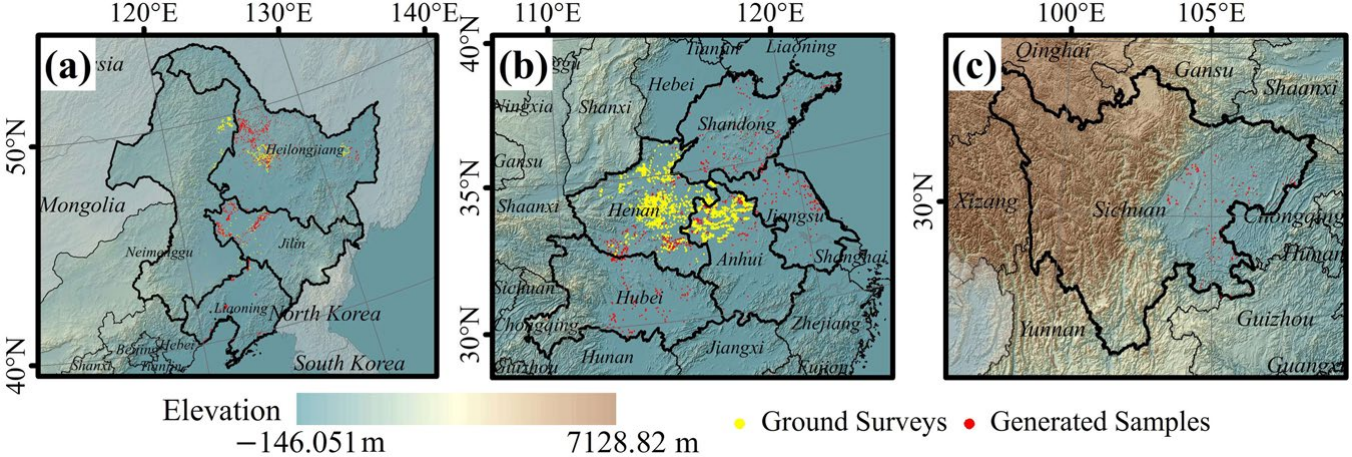
Location of the major soybean-producing region in (**a**) Northeast China, (**b**) Huang-Huai-Hai Plain and the Middle-Lower Yangtze Plain, and (**c**) Sichuan Basin.

This study aimed to map the distribution of the soybean planting areas from 2019 to 2022 in ten provinces, namely Heilongjiang, Inner Mongolia, Anhui, Sichuan, Henan, Jilin, Jiangsu, Shandong, Hubei, and Liaoning (Fig. 2). These provinces are recognized as the top soybean-producing regions in China, collectively accounting for more than 80% of soybean production^3^. To effectively map the soybean annual planting area in China, we categorized them into three main regions: (a) Northeast China, encompassing Heilongjiang, Jilin, Liaoning, and the northeastern Inner Mongolia; (b) Huang-Huai-Hai Plain and the Middle-Lower Yangtze Plain, covering Shandong, Henan, Anhui, Jiangsu, and Hubei; and (c) Sichuan Basin.

### Data

The Sentinel-2 satellite, with a spatial resolution of 10 meters and a revisiting period of 5 days, offers optimal support for the comprehensive and long-term identification of crops. Equipped with the Multispectral Instrument (MSI), Sentinel-2 can effectively even the slightest variations between different crops. For our study area, we acquired all available Sentinel-2A/B (S2) Level-2A surface reflectance (SR) data from 2019 to 2022 through the Google Earth Engine (GEE) platform. To enhance data quality, cloud masking and Savitzky-Golay (SG) filtering techniques were applied to the acquired data.

Two categories of spectral data were utilized to classify soybeans and other crop types: (1) reflectance from five spectral bands and (2) the values of nine spectral indices (refer to Table 1). The five bands selected for classification are red edge 1 (RE1), red edge 2 (RE2), red edge 3 (RE3), shortwave infrared 1 (SWIR1) and shortwave Infrared 2 (SWIR2). The red-edge bands are important indicator bands that reflect plant pigments and health status. The short-wave infrared bands can reflect changes in moisture and other biochemical components in crop leaves^42^. Previous research has confirmed the significant role they play in distinguishing between soybeans and corn^13,20,43^. Additionally, nine commonly employed spectral indexes were computed: Enhanced Vegetation Index (EVI)^44^, Green Chlorophyll Vegetation Index (GCVI)^45^, Land Surface Water Index (LSWI)^46^, Red Edge Position Index (REPI)^47^, Red Edge Normalized Difference Vegetation Index (RENDVI)^48^, Normalized Difference Phenology Index (NDPI)^49^, and Soil-Adjusted Vegetation Index (SAVI)^50^, Optimized Soil-Adjusted Vegetation Index (OSAVI)^51^, Transformed Chlorophyll Absorption in Reflectance Index (TCARI)^52^. The use of NDVI and EVI time series is widespread for extracting temporal characteristics and phenological indicators of various crops. LSWI can effectively differentiates and classifies rice due to the heightened responsiveness of corn and soybeans to leaf and soil moisture. RENDVI and REPI, which leverage the S2 Red Edge bands, are particularly suited for estimating canopy chlorophyll II and nitrogen content. OSAVI proficiently mirrors the dynamic growth of crops while simultaneously minimize the impact of background soil^51^. There exists a high correlation between crop OSAVI and their canopy chlorophyll content, which displays significant variations throughout the growth season of the crops. In crops with high chlorophyll content, such as soybeans, corn, and rice, changes in TCARI are comparatively slow. Therefore, TCARI/OSAVI demonstrates considerable sensitivity to flux in chlorophyll content^52^. Cash crops such as peanuts, cotton, potatoes, and sunflowers, potentially outside of soybeans, are derived using TCARI/OSAVI^25^.

**Table 1:** Formulas of nine spectral indices used in the study.

Indices	Formulation*
EVI	${\rm{EVI}}=2.5\times \frac{{{\rm{\rho }}}_{NIR}-{{\rm{\rho }}}_{red}}{{{\rm{\rho }}}_{NIR}+6\times {{\rm{\rho }}}_{red}-7.5\times {{\rm{\rho }}}_{blue}+1}$
GCVI	${\rm{GCVI}}=\frac{{{\rm{\rho }}}_{NIR}}{{{\rm{\rho }}}_{green}}-1$
LSWI	${\rm{LSWI}}=\frac{{{\rm{\rho }}}_{NIR}-{{\rm{\rho }}}_{SWIR1}}{{{\rm{\rho }}}_{NIR}+{{\rm{\rho }}}_{SWIR1}}$
REPI	${\rm{REPI}}=705+35\times \frac{\left({{\rm{\rho }}}_{red}+{{\rm{\rho }}}_{RE3}\right)/2-{{\rm{\rho }}}_{RE1}}{{{\rm{\rho }}}_{RE2}-{{\rm{\rho }}}_{RE1}}$
RENDVI	${\rm{RENDVI}}=\frac{{{\rm{\rho }}}_{NIR}-{{\rm{\rho }}}_{RE2}}{{{\rm{\rho }}}_{NIR}+{{\rm{\rho }}}_{RE2}}$
NDPI	${\rm{NDPI}}=\frac{{{\rm{\rho }}}_{{\rm{NIR}}}-\left(0.78\times {{\rm{\rho }}}_{{\rm{red}}}+0.22\times {{\rm{\rho }}}_{{\rm{SWIR1}}}\right)}{{{\rm{\rho }}}_{{\rm{NIR}}}+\left(0.78\times {{\rm{\rho }}}_{{\rm{red}}}+0.22\times {{\rm{\rho }}}_{{\rm{SWIR1}}}\right)}$
SAVI	${\rm{SAVI}}=\frac{{{\rm{\rho }}}_{{\rm{NIR}}}-{{\rm{\rho }}}_{{\rm{red}}}}{{{\rm{\rho }}}_{{\rm{NIR}}}+{{\rm{\rho }}}_{{\rm{red}}}+0.5}\times 1.5$
OSAVI	${\rm{OSAVI}}=\frac{1.16\times \left({{\rm{\rho }}}_{NIR}-{{\rm{\rho }}}_{red}\right)}{\left({{\rm{\rho }}}_{NIR}+{{\rm{\rho }}}_{SWIR1}+0.16\right)}$
TCARI	${\rm{TCARI}}=3\times \left(\left({{\rm{\rho }}}_{{\rm{RE}}1}-{{\rm{\rho }}}_{{\rm{red}}}\right)-0.2\times \left({{\rm{\rho }}}_{{\rm{RE}}1}-{{\rm{\rho }}}_{{\rm{green}}}\right)\times \frac{{{\rm{\rho }}}_{{\rm{RE}}1}}{{{\rm{\rho }}}_{{\rm{red}}}}\right)$

^*^ρ_*blue*_*, ρ*_*green*_*, ρ*_*red*_*, ρ*_*RE*1_*, ρ*_*RE*2_*, ρ*_*RE*3_*, ρ*_*NIR*_ *and ρ*_SWIR1_, is surface reflectance of Band 2 (blue, 496.6 nm (S2A)/492.1 nm (S2B)), Band 4 (red, 664.5 nm (S2A)/665 nm (S2B)), Band 5 (Red Edge 1, 703.9 nm (S2A)/703.8 nm (S2B)), Band 6 (Red Edge 2, 740.2 nm (S2A)/739.1 nm (S2B)), Band 7 (Red Edge 3, 782.5 nm (S2A)/779.7 nm (S2B)), Band 8 A (NIR, 864.8 nm (S2A)/864 nm (S2B)), Band 11 (SWIR1, 1613.7 nm (S2A)/1610.4 nm (S2B)) in the Sentinel-2 MSI sensor.

The availability of suitable Sentinel-2 images was limited due to frequent cloud cover and rain during the soybean growing season. This presented challenges in creating the necessary time-series spectral features for classification. To address this issue, Sentinel-1 SAR (Synthetic Aperture Radar) data was utilized to establish the required time-series spectral features for classification. Sentinel-1 is equipped with a C-band synthetic aperture radar operating at a center frequency of 5.045 GHz. It provides four imaging modes: Stripmap, Interferometric Wide swath, Extra Wide swath, and Wave modes. Sentinel-1 offers dual-polarization SAR data (HH+HV, VV+VH) and has four product specifications: RAW Level-0, SLC (Single-Look Complex), GRD (Ground Range Detected), and OCN (Ocean). Due to data storage limitations, Sentinel-1 images in GEE are accessible in the GRD format, which lacks phase information. The data in GEE undergo several pre-processing steps, which include: 1) removal of thermal noise, 2) radiometric calibration, 3) terrain correction using SRTM or ASTER DEM data, and 4) conversion of terrain-corrected backscattering coefficients to decibel values. Given the potential adverse effects of SAR active microwave imaging on image quality, this study applied Refine Lee filtering and straightforward incidence angle normalization into the processing of Sentinel-1 images.

The present study employs Sentinel-1 VV/VH dual-polarization imagery to distinguish five SAR parameters for extracting soybean features. These parameters comprise the backscattering ratio for VV and VH, denoted as ${{\rm{\sigma }}}_{{\rm{VH}}}^{{\rm{0}}}$ and ${{\rm{\sigma }}}_{{\rm{VV}}}^{{\rm{0}}}$ respectively, in addition to three combinations of polarization channels: the cross-polarization ratio (${{\rm{\sigma }}}_{{\rm{VH}}}^{{\rm{0}}}{{\rm{/\sigma }}}_{{\rm{VV}}}^{{\rm{0}}}$), the cross-polarization sum (${{\rm{\sigma }}}_{{\rm{VH}}}^{{\rm{0}}}{{\rm{+\sigma }}}_{{\rm{VV}}}^{{\rm{0}}}$), and the Radar Vegetation Index (RVI). These parameters are itemized in Table 2.

**Table 2:** The five SAR-based features used in the study.

Features	Proxies	Description
backscattering ratio	${{\rm{\sigma }}}_{{\rm{VH}}}^{{\rm{0}}}{{\rm{,\sigma }}}_{{\rm{VV}}}^{{\rm{0}}}$	Throughout the soybean growth period, alterations in the growth status and density of soybean leaves, stems, and pods can have substantial effects on the backscattering ratio^67^.
cross-polarization ratio	${{\rm{\sigma }}}_{{\rm{VH}}}^{{\rm{0}}}{{\rm{/\sigma }}}_{{\rm{VV}}}^{{\rm{0}}}$	Fluctuations over time in this index reflect changes in moisture content and structure that are associated with phenological development^68^.
cross-polarization sum	${{\rm{\sigma }}}_{{\rm{VH}}}^{{\rm{0}}}{{\rm{+\sigma }}}_{{\rm{VV}}}^{{\rm{0}}}$	Cross-polarization is highly correlated with crop Leaf Area Index (LAI) and crop height^69^.
RVI	${\rm{RVI\; =}}\frac{{\rm{4}}\times {{\rm{\sigma }}}_{{\rm{VH}}}^{{\rm{0}}}}{{{\rm{\sigma }}}_{{\rm{VH}}}^{{\rm{0}}}{{\rm{+\sigma }}}_{{\rm{VV}}}^{{\rm{0}}}}$	RVI can characterize both crop biomass and the LAI^70^.

### Training and validation data

Ground survey samples, include those we collected from various provinces across different years, are denoted as yellow points in Fig. 2 and list in Table 3. The sample locations and crop types were recorded during fieldwork using mobile Geographic Information System (GIS) devices. Post-field surveys, we conducted a visual inspection of all ground samples utilizing high-resolution images from Google Earth and Sentinel-2 RGB composite images. Any samples displaying evident errors, such as the misclassification of natural vegetation as crops, were discarded. Samples located close to roads or field boundaries were also excluded. In addition, the sample data was enhanced by using existing data products^53^.

**Table 3:** The number of soybean samples collected by ground survey in different provinces and years.

Province	2019	2020	2021	2022
Heilongjiang	1737	500	—	226
Jilin	141	3694	—	—
Liaoning	183	422	—	—
Inner Mongolia	825	—	1393	—
Henan	—	1967	—	—
Anhui	919	341	—	—
Chongqing	—	—	—	564

### Sample generation and migration

**Fig. 3:**
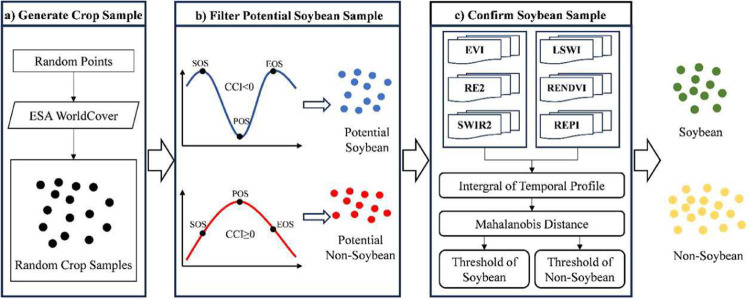
Sample generation process. SOS, start of growing season; POS, peak of growing season; EOS, end of growing season; CCI, Concave-Convexity Index.

This paper introduces a method for generating samples that employs existing samples to facilitate the spatiotemporal migration of soybean samples, even amidst constraints in sample sizes and temporal coverage (Fig. 3). The strategy used in this study for generating samples involves sifting out soybean and non-soybean specimens from randomly collected cropland samples. In our study area, the primary crops grown encompass soybeans, corn, rice, wheat, and other staple crops, the planting area of which comprises up to 62% of the total cultivated land area^3^. Peanuts, rapeseed, cotton, potatoes, sunflowers, and other cash crops are also cultivated. The production of winter wheat and winter rapeseed in China accounts for more than 90% of the total wheat and rapeseed production, respectively^54^. As the growth periods of these two do not overlap with that of soybeans, they are not considered when filtering sample. To distinguish soybeans from the aforementioned non-soybean samples, our method of generating samples is categorized into three parts. Initially, the ESA WorldCover^55^ is used to generate random cropland samples with unspecified crop types. Subsequently, based on the findings of Huang *et al*.^25^, we devised the Concave-Convexity Index (CCI), which segregates random crop samples into potential soybeans and non-soybeans based on the chlorophyll content change in the crop canopy. By charting the time series curves of band reflectivity and crop vegetation indexes, it is possible to discern accurate and reliable samples from potential ones, achieved through the analysis of the typical distribution of the area under the curve .

**Fig. 4:**
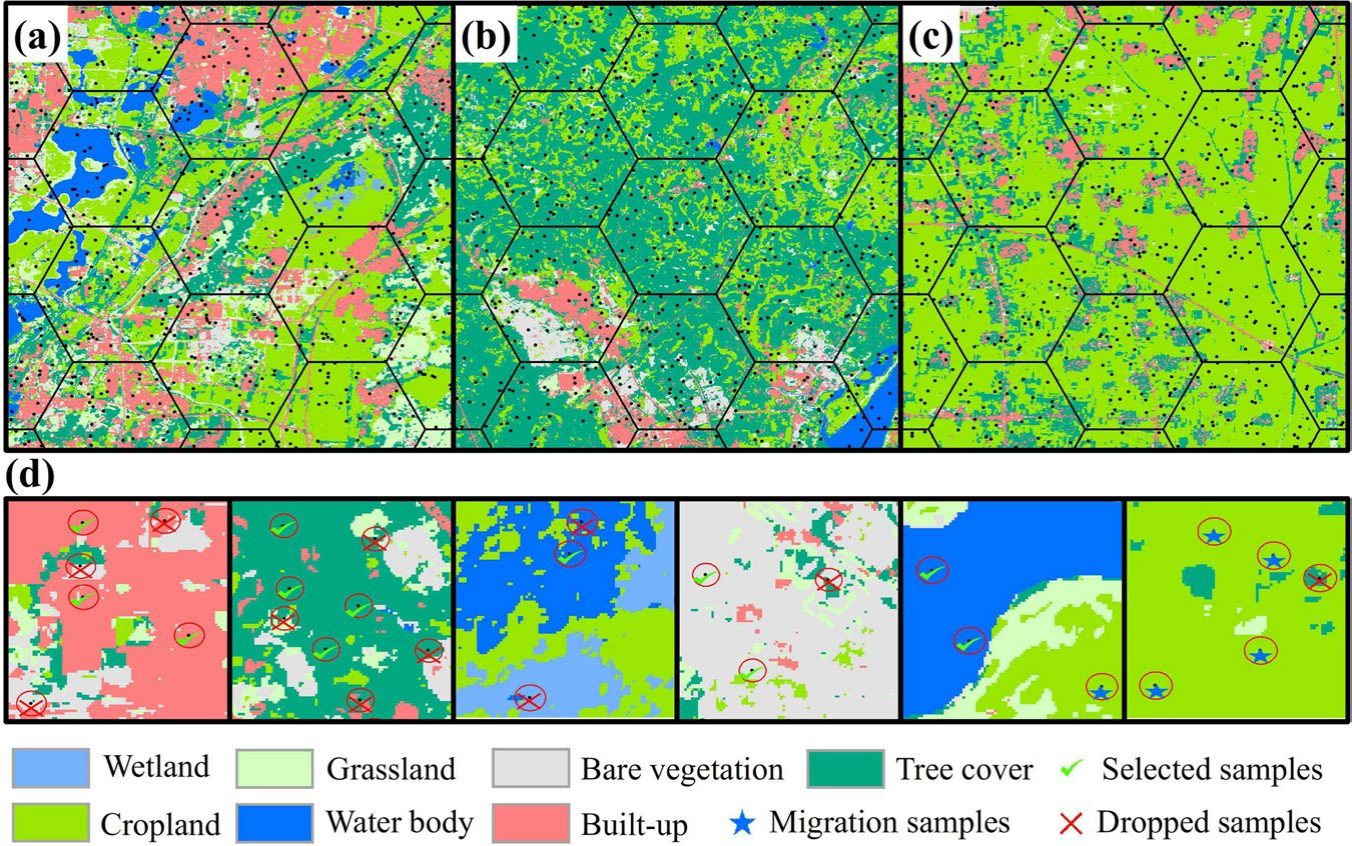
Grid-based random sample point generation with ESA WorldCover on the base map, green checkmarks indicate that a sample point of that feature type is retained, red fork markers indicate that the sample is discarded, and blue star markers indicate that cropland samples are retained for subsequent sample generation.

**Fig. 5:**
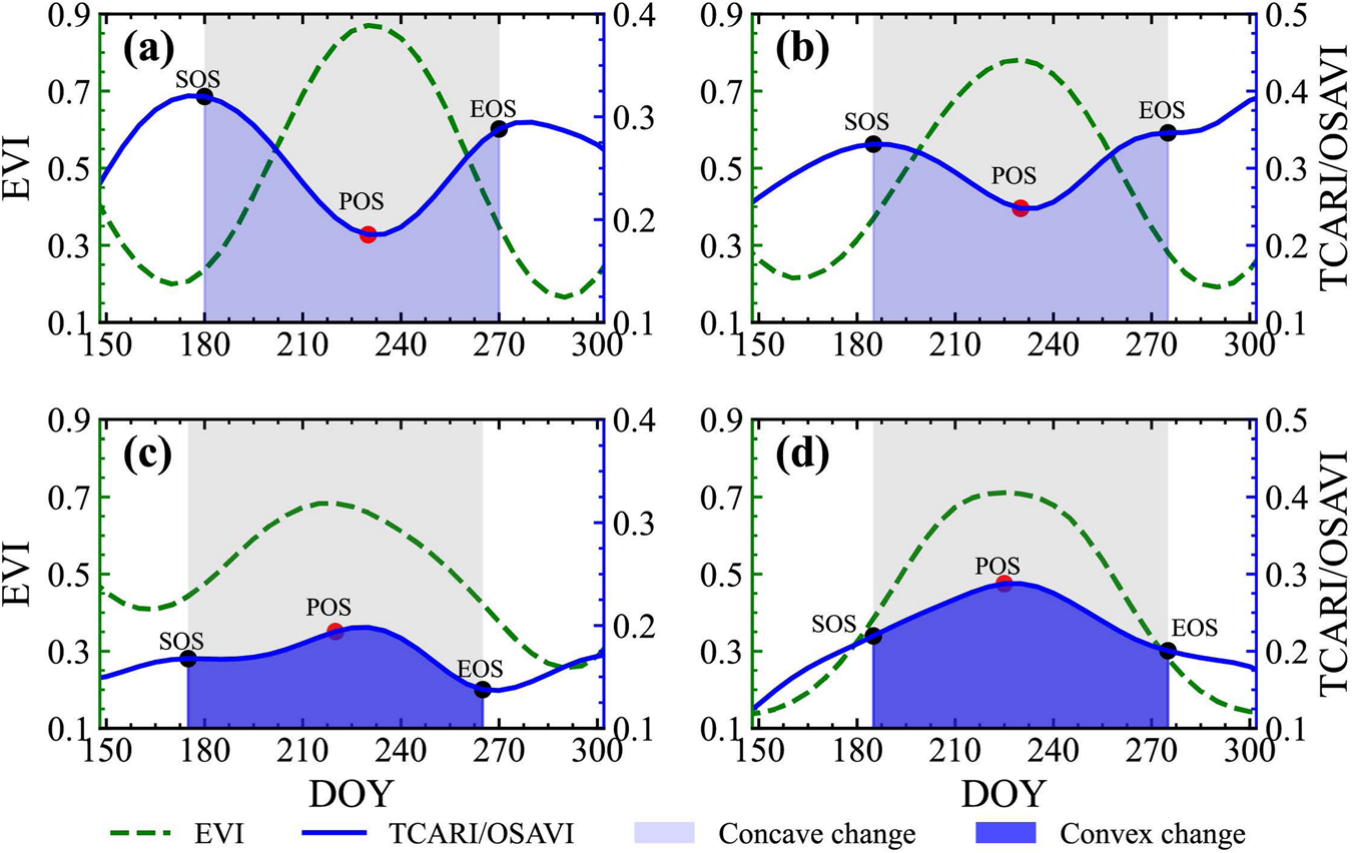
Temporal profile of TCARI/OSAVI for (**a,b**) soybeans and (**c,d**) other crops (including peanuts, cotton, and potatoes).

**Fig. 6:**
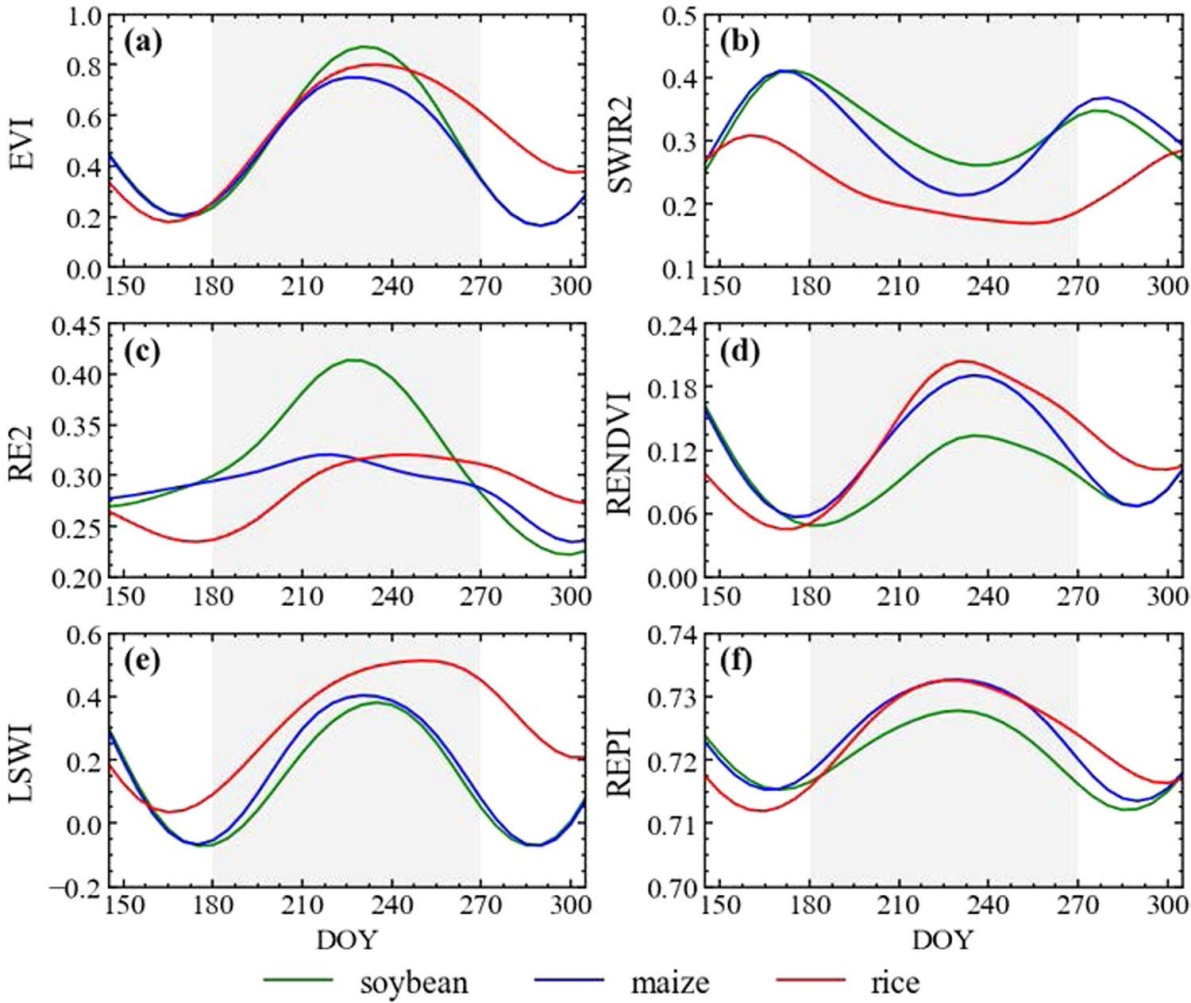
Time-series vegetation index curves for soybeans and other major crops.

- **Generate crop samples**. Different forms of land cover, including tree cover, grassland, water bodies, and buildings, are often proximate to agricultural areas. These elements may influence the integrity of the crop sample, as illustrated in Fig. 4. In our approach, a hexagonal automated sampling technique is utilized to generate random crop points^56^, which involves scattering a substantial number of points randomly within a hexagonal grid and determine the point’s classification by appraising the proportion of land cover categories within a 50-meter buffer. If a single land cover type comprises more than 90% of the area within this buffer, it is assigned as the type for that point. To verify the accuracy of samples for uncultivated area, we performed visual analysis using high-resolution Google Earth images and Sentinel-2 data from the corresponding year.
- **Filter potential soybean samples**. We initiated a preliminary filtering process of random crop points, aimed at identifying potential soybeans and non-soybeans. Crops’ OSAVI is highly correlated with their canopy chlorophyll content, showing significant variations as the crops grow^51^. In crops with high chlorophyll content, such as soybeans, corn, and rice, TCARI changes relatively gradually^52^. As a result, the TCARI/OSAVI trend typically displays a concave pattern in these crops^25^. Conversely, crops with low chlorophyll content, such as peanuts, cotton, potatoes and sunflowers exhibit an inverse pattern, marked by noteworthy alterations in TCARI and culminating in a convex temporal curve, as illustrated in Fig. 5. This study leverages the TCARI/OSAVI association to evaluate the concavity or convexity of the temporal growth curves of crops by establishing the CCI for the start of growing season (SOS), peak of growing season (POS), and end of growing season (EOS)^25^. Points exhibiting a CCI ≥ 0 are marked as potential non-soybean points, while those with CCI < 0 are earmarked as possible soybean points. These points are subsequently employed in the sample selection process. The dates when the EVI temporal curve attains its peak are deemed as the POS of the crop, while the SOS and EOS are calculated using the median method^57^. The formulation for CCI calculation (Eq. 1) is provided below:
  1 $$CCI=2\times {\left(\frac{TCARI}{OSAVI}\right)}_{POS}-\left[{\left(\frac{TCARI}{OSAVI}\right)}_{SOS}+{\left(\frac{TCARI}{OSAVI}\right)}_{EOS}\right]$$
- **Confirm soybean samples**. Several time series curves of band reflectivity and vegetation indexes, accurately exhibiting the growth characteristics of soybeans, as presented in Fig. 6. The curve integration of a specific parameter represents its cumulative value throughout the entire crop growing season. Soybeans, during their peak growth phase, manifest increased dryness and greener foliage, distinguishing them from other crops^27^. Consequently, soybeans tend to exhibit elevated values for EVI and SWIR2, while their LSWI values are comparatively lower. During the peak growing season, the REPI and RENDVI for corn distinctly surpass those of soybeans^20^. Conversely, soybeans demonstrate a relatively high red edge reflectance. Accordingly, we formulated two parameter sets for soybean samples screening, which includes a high-value group (EVI, RE2, and SWIR2; see Fig. 6a–c), and a low-value group (LSWI, RENDVI, and REPI; see in Fig. 6d,e). As a result, soybeans can be clearly distinguished from other crops through curve integration. The integration limits align with the crop’s SOS and EOS.

The band reflectivity or vegetation index of a certain crop in the peak growing season follows a one-dimensional Gaussian distribution ^32^. The integral values of the aforementioned two sets of time series curves of soybeans are assumed to follow a multivariate Gaussian distribution that is connected to their dimensions. The probability density function (Eq. 2) is given by:

2 $$PDF\left(x\right)=\frac{1}{{\left(\sqrt{2\pi }\right)}^{3}{\left|\Sigma \right|}^{\frac{1}{2}}}\exp \left(-\frac{1}{2}{\left(x-\mu \right)}^{T}{\Sigma }^{-1}\left(x-\mu \right)\right)$$

Where *x* is the integral vector of time series curve from ether the high-value or low-value group, *μ* is the mean vector of the verified soybean points, and Σ is the covariance matrix. To quantify the resemblance between random crop points and verified soybean points, we employed the Mahalanobis distance measurement. This computation is performed between the randomly selected points and a multivariate Gaussian distribution, which is composed of verified soybean points. The Mahalanobis distance gauges the deviation between data points and distributions, factoring in the correlation among different dimensions. This method mitigates the effects of varying dimensions and variances, thereby yielding a more accurate representation of the correlation between two sets of data. The Mahalanobis distance (Eq. 3) for a multivariate vector, with a mean of *μ* and a covariance matrix Σ is defined as follows:

3 $${D}_{M}\left(x,\mu \right)=\sqrt{{\left(x-\mu \right)}^{T}{\Sigma }^{-1}\left(x-\mu \right)}$$

**Fig. 7:**
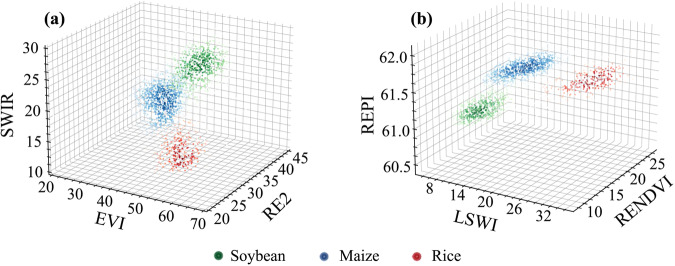
Spatial Distribution of Soybean Features, (**a**) High-Value Group, (**b**) Low-Value Group.

Figure 7 depicts the scatter distributions of soybeans, corn, and rice in three-dimensional spaces for high-value and low-value groups. Three crops clearly form distinct groups. The soybean samples are clustered in the upper-right and lower-left corners of two feature spaces. This highlights the excellent ability of the area under the curve employed in this study to distinguish soybeans from other crops. In the previous sections, we computed two sets of Mahalanobis distances between filtered points and points representing soybeans from ground survey. We hypothesize that shorter distances are indicative of soybeans, therefore emphasising the need to determine the categorization threshold. The threshold is determined by analysing the multivariate Gaussian distributions using soybean samples as the basis. Points with a high probability density are indicative of the most salient features of soybeans, and these points are concentrated at the centre of the distribution. To identify these robust soybean points, we employed the Monte Carlo method to calculate the probability density *p*_50_ at which the cumulative probability of the multivariate Gaussian distribution (which is symmetric around the centre) hits 50%. The sought-after points are those soybean points with a probability density higher than *p*_50_. The formula (Eqs. 4, 5) for *p*_50_ is given as:

4 $$\underset{{\boldsymbol{\mu }}-{\boldsymbol{t}}}{\overset{{\boldsymbol{\mu }}+{\boldsymbol{t}}}{\int }}PDF\left(x\right)dx=50{\rm{ \% }}$$

5 $${p}_{50}=PDF\left(\mu -t\right)$$

Subsequently, we computed the Mahalanobis distances from robust soybean points to the multivariate Gaussian distributions of high-value group and low-value group. The 90th percentile of these distances was selected as the threshold for confirming reliable soybean points from potential soybean, namely ${D}_{Soy-Thresh-High}$ and ${D}_{Soy-Thresh-Low}$. For non-soybean points, we determined the 95th percentile of Mahalanobis distances from all ground survey soybean samples to the distributions, establishing ${D}_{NonSoy-Thresh-High}$ and as filtering thresholds. The filtering targets include potential soybean and non-soybean. Ultimately, the criteria (Eqs. 6, 7) for selecting soybean and non-soybean points from random points are as follows:

6 $$\begin{array}{l}if\left(CCI < 0\right)and\left({D}_{High} < {D}_{Soy-Thresh-High}\right)and\left({D}_{low} < {D}_{Soy-Thresh-Low}\right),\\ Soybean=1;else,Soybean=0\end{array}$$

7 $$\begin{array}{l}if\left({D}_{High} > {D}_{NonSoy-Thresh-High}\right)and\left({D}_{low} > {D}_{NonSoy-Thresh-Low}\right),\\ Non-Soybean=1;else,Non-Soybean=0\end{array}$$

Where *D*_*High*_ and *D*_*Low*_ denote the Mahalanobis distances between arbitrary points and the high-value and low-value classes of soybean ground surveys, respectively. Figure 7 depicts the ellipsoidal clustering pattern of soybean points within a three-dimensional feature space. This study shows that the robust soybean points extracted through probability density occupy the central region of the ellipsoid. The percentiles of Mahalanobis distance from these points to the distribution guarantee that the filtered soybean points are also located within the ellipsoid, thereby assuring their accuracy and reliability. It is noteworthy that the computation of Mahalanobis distance involves dimension independence and standardization, which convert the ellipsoid into a sphere in the Mahalanobis distance space, thereby expanding the precision of the point filtering process.

**Fig. 8:**
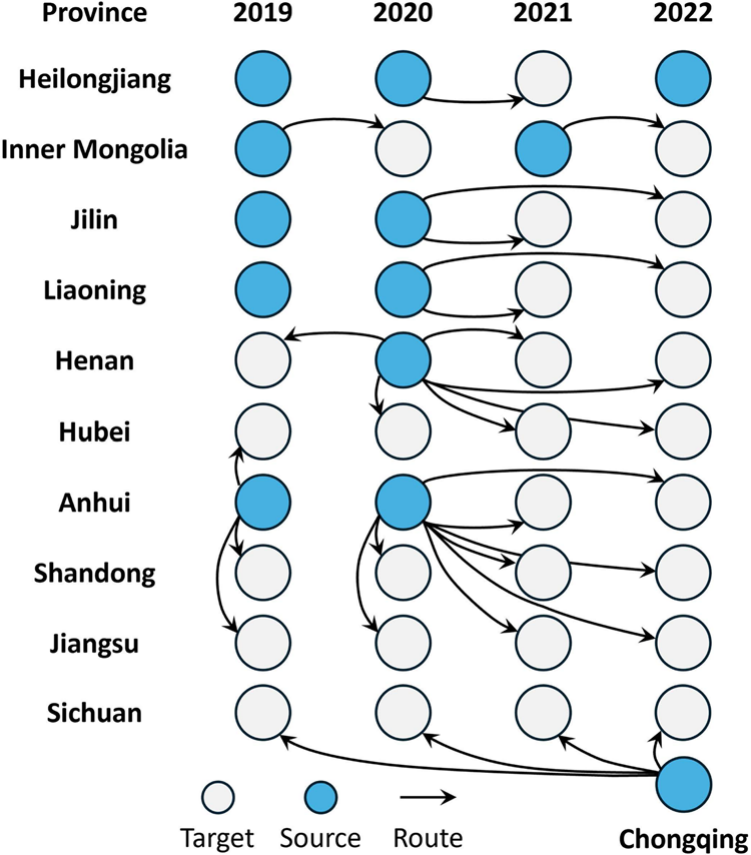
Ground survey samples used for samples migration in each province and year.

In terms of migration strategy, we categorized the strategy into three types based on the spatiotemporal relationship between ground survey samples and migrated ones. These categories include: (1) Migrating samples from different regions in the same year to the target area (spatial migration); (2) Migrating samples from different years in the same region to the target year (temporal migration); (3) Migrating samples from different years and regions to the target year in the intended region (spatiotemporal migration). We give priority to using ground survey samples from the same region for migration (temporal migration), because soybean in the same area have relatively little inter-annual changes in planting habits, varieties, and soil texture^58^. Therefore, we think the temporal heterogeneity of soybeans in spectral parameters is smaller than the spatial heterogeneity. If there are no ground surveys in a certain area, samples from similar climate zones will be used for migration, with priority given to those from the same years, then adjacent years. Figure 8 describes the provinces and years to which the ground survey samples used for sample migration belong.

To evaluate the effectiveness of sample generation, we employed the GLAD maize and soybean map (GLAD)^13^ to assess the accuracy of the generated soybean and non-soybean samples. Since the timeline of GLAD is constrained to 2019, we performed experiments and computed accuracies only for the sample points generated in 2019, implying all target years are set as 2019.

### Features selection and classification

We utilized soybean phenological characteristics and spectral indexes to differentiate between soybean and non-soybean crops, as these are efficient in capturing the seasonal fluctuations in surface spectra. To compile these indicators, we annually selected data from April 1^st^ to November 15^th^, considering the crop calendars of various regions. Table 4 presents the candidate features we employed. Statistical features of five reflectance bands - RE1-3, SWIR1, and SWIR2 - were analysed during the growing season (DOY: 90–318). These encompass the minimum, maximum, and standard deviation, as well as the 15th, 50th, and 90th percentiles. Phenological parameters obtained from the EVI time series, harmonic fitting parameters^20,59^, and accumulative biomass attributes^60,61^ are also taken into account. We utilized harmonic fitting (discrete Fourier transform, Eq. 8) analysis to the original effective observational data to extract time-series curves, as demonstrated in the following formula:

8 $$f\left(t\right)=a+b\times t+\mathop{\sum }\limits_{m=1}^{M}\left({C}_{M}\cos \left(2\pi \omega t\right)+{D}_{M}\sin \left(2\pi \omega t\right)\right)+e$$

Where *f*(*t*) represents fitted vegetation index value at the time instance *t*. The constant term is represented as *a*, while *b* corresponds to the coefficient of the first-order term. *M* signifies the quantity of harmonic components, and *C* and *D* stand for the coefficients of cosine and sine functions, respectively. The variable *ω* is the reciprocal of the number of days in a year (1/365), *t* represents a specific day within a year as denoted by the DOY, and *e* corresponds to the residual value. For temporal feature extraction, phase and amplitude are utilized with amplitude defined as the magnitude of a two-dimensional vector [*C*_*M*_,*D*_*M*_], and phase as the angle of the same two-dimensional vector [*C*_*M*_,*D*_*M*_].

**Table 4:** Summary of Optical Feature Parameters and Feature Extraction Methods.

Feature Type	Feature Name	Processing Method	Quantity
Vegetation Indices Time Series	EVI, GCVI, LSWI, REPI, NDPI	min, max, std, and 15/50/90th percentile	5 × 6
Red-Edge Band Time Series	B5, B6, B7 $\left(704nm-782nm\right)$		3 × 6
Shortwave Infrared Band Time Series	B11, B12 $\left(1610nm-2200nm\right)$		2 × 6
Phenological Features	SOS EOS LOS	Median method	3 × 1
EVI Time Series Features	(EVI) Phase and Amplitude	Harmonic fitting	2 × 2
Accumulated Biomass Features	EVI	Accumulation	2 × 1

In assessing biomass using EVI, the EVI is systematically calculated for every time series data point throughout the growth season. The subsequent step involves aggregating the EVI values over unique time intervals to derive the cumulative biomass features, a crucial factor for soybean classification. To accommodate potential inconsistencies stemming from diverse pixel observation frequencies in regions with incomplete image data, a trilinear interpolation approach is incorporated in this study. This method effectively corrects missing data points, thereby ensuring a uniform computation of cumulative biomass features. The formula for trilinear interpolation (Eq. 9) is provided below:

9 $$\begin{array}{l}{y}_{0}={y}_{2}+\frac{f\left[{x}_{2},{x}_{2},{x}_{3}\right]{\left({x}_{0}-{x}_{2}\right)}^{2}+f\left[{x}_{1},{x}_{2},{x}_{3}\right]\left({x}_{0}-{x}_{2}\right)\left({x}_{0}-{x}_{3}\right)}{\left({x}_{2}-{x}_{1}\right)\left({x}_{2}-{x}_{3}\right)}\\ \quad \;\;\;\;\;\;+\frac{f\left[{x}_{1},{x}_{2},{x}_{2}\right]{\left({x}_{0}-{x}_{2}\right)}^{2}+f\left[{x}_{1},{x}_{2},{x}_{3}\right]\left({x}_{0}-{x}_{1}\right)\left({x}_{0}-{x}_{2}\right)}{\left({x}_{3}-{x}_{2}\right)\left({x}_{2}-{x}_{1}\right)}\end{array}$$

Within this context, *y*_0_ denotes the sought-after interpolation outcome, with *y*_2_ denoting known function values. Here, *x*_0_ designates the point slated for interpolation, whereas *x*_1_, *x*_2_, and *x*_3_ stand as the abscissas for three established reference points. The notation *f*[*x*_1_,*x*_2_,*x*_3_] represents the third-order divided difference calculated at positions *x*_1_, *x*_2_, and *x*_3_.

In addressing the five SAR feature parameters (Table 5), we harnessed SAR image time series to extract pivotal phenological characteristics of crops. This process comprised both statistical features and principal component features. The statistical features mirrored the approach adopt for optical data, incorporating the maximum, minimum, and variance, along with the 15th, 50th, and 90th percentiles of the five SAR parameters. These statistical attributes are instrumental in conveying the average levels and temporal fluctuations within time series curves for diverse crops. Furthermore, we carried out a Principal Component Analysis (PCA) on the Sentinel-1 image time series in the temporal domain, selecting the initial three principal components as the principal component features of SAR data^62^.

**Table 5:** Summary of SAR Feature Parameters and Feature Extraction Methods.

Feature Type	Feature Name	Processing Method	Quantity
Statistical Features	${{\rm{\sigma }}}_{{\rm{VH}}}^{{\rm{0}}},{{\rm{\sigma }}}_{{\rm{VV}}}^{{\rm{0}}},{{\rm{\sigma }}}_{{\rm{VH}}}^{{\rm{0}}}+{{\rm{\sigma }}}_{{\rm{VV}}}^{{\rm{0}}},\frac{{{\rm{\sigma }}}_{{\rm{VH}}}^{{\rm{0}}}}{{{\rm{\sigma }}}_{{\rm{VV}}}^{{\rm{0}}}},{\rm{RVI}}$	max, min, mean, stdv, 15/50/90th percentile	7 × 5
Principal Component Features	${{\rm{\sigma }}}_{{\rm{VH}}}^{{\rm{0}}},{{\rm{\sigma }}}_{{\rm{VV}}}^{{\rm{0}}},{{\rm{\sigma }}}_{{\rm{VH}}}^{{\rm{0}}}+{{\rm{\sigma }}}_{{\rm{VV}}}^{{\rm{0}}},\frac{{{\rm{\sigma }}}_{{\rm{VH}}}^{{\rm{0}}}}{{{\rm{\sigma }}}_{{\rm{VV}}}^{{\rm{0}}}},{\rm{RVI}}$	Principal Component Analysis	3 × 5

Considering the ‘Hughes’ Phenomenon, the current number of features is copious. Consequently, in each classification process, we incorporate feature selection, choosing to maintain the top 50% of features. This decision is influenced by the ranking provided by the random forest model for the final classification.

We employed local random forest classifiers for soybean planting areas identification in each province. This non-parametric machine learning classifier exhibits a higher error tolerance compared to certain parametric classifiers and has been extensively utilized in classification and recognition research. In terms of dividing training set and testing set, half of the samples within each province were randomly selected for training the classifier and mapping soybean cropland, while the remainder were utilized for validation. In this study, we implement the random forest classification model within the GEE. On the GEE platform, we vary the number of decision trees from 50 to 500 at 50-unit intervals. The chosen number of decision trees as the parameter for ensuring classification is the one that surpasses 100 and achieves the initial local maximum in classification accuracy. To counteract minor result variations in each experimental repetition due to the inherent randomness in random forest sampling, we set a random seed of 999. All additional parameters are left at their default values.

To assess the precision of soybean distribution mapping, we take two approaches: (1) on-site validation through the collection of ground truth samples, which involves conducting ground surveys and generating samples, and (2) comparing the results with agricultural statistical data obtained from administrative units. Confusion matrices were generated using both soybean samples and non-soybean samples for each provincial soybean map. These matrices were employed to calculate the producer’s accuracy (PA), user’s accuracy (UA) and F1-score (F1) for soybean samples (Eqs. 10–12), assessing the precision of the approaches. The overall success of this strategy was assessed by calculating the overall accuracy (OA). The Kappa coefficient was used to assess the level of agreement between the classification results and sample labels. In addition, we assessed the soybean planting area identified in this study by comparing it to agricultural statistical data at the provincial and prefectural levels. This comparison was done using the coefficient of determination (R2), root mean square error (RMSE), and mean absolute error (MAE).

10 $$PA=\frac{TP}{TP+FP}$$

11 $$UA=\frac{TP}{TP+FN}$$

12 $$F1=2\times \frac{PA\times UA}{PA+UA}$$

### Post-processing

For large-scale and high-resolution crop mapping, the speckle noise is inevitable, and the same goes for soybean mapping^63^. Errors may arise during sensor imaging, soybean sample generation, image preprocessing, and feature classification, etc., resulting in soybean patches composed of just one or two pixels in the mapping results. In most cases, they are considered speckle noise and should be eliminated. We performed post-processing on the results using eight-neighborhood majority filtering. This processing can filter independent, unconnected soybean pixels, and non-soybean pixels in soybean plots will also be filled, making the mapping results more accurate and reasonable.

## Data Records

Between 2019 and 2022, we generated four soybean cropland maps encompassing China’s key soybean-producing regions, all at a 10-meter spatial resolution (ChinaSoybean10). The datasets, formatted to Geotiff, are available for access at the Zenodo repository (10.5281/zenodo.10068402)^64^. Structured under the ESPG: 4326 (WGS_1984) spatial reference system, the maps incorporate only one values: 1 to denote soybean planting areas, and null value to indicate non-soybean planting areas (inclusive of other landcover). These maps can be scrutinized and visualized using software such as ArcGIS, QGIS, or their alternatives.

## Technical Validation

### Precision Assessment of Sample Spatiotemporal Migration

**Fig. 9:**
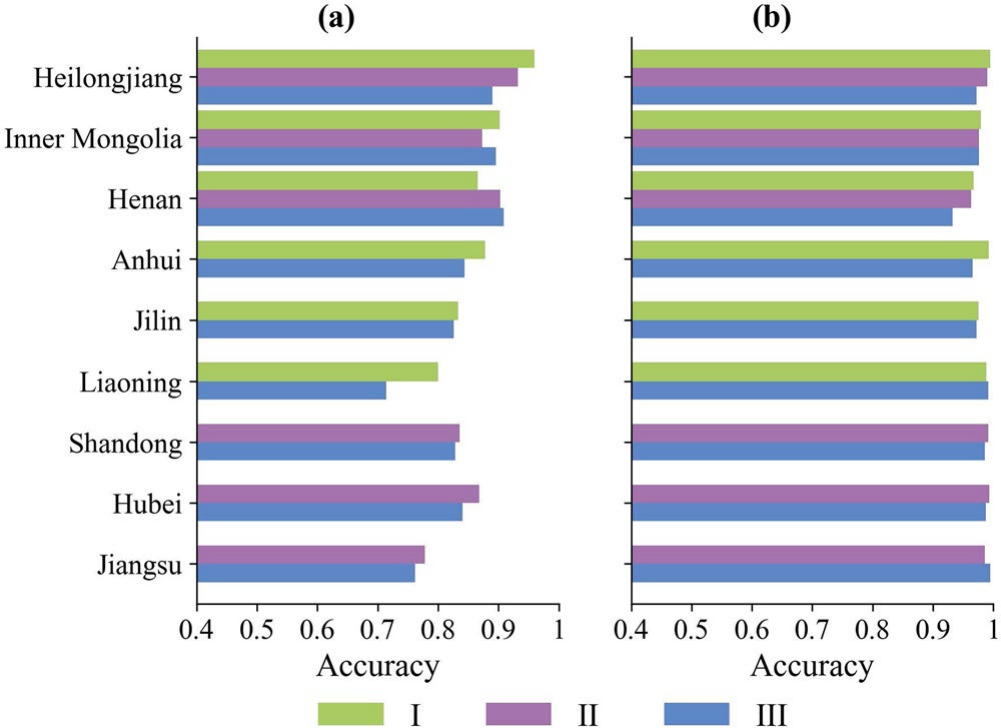
2019 generated sample precision evaluation, (**a**) Soybeans, (**b**) Non-Soybeans, I-III represents different methods for sample migration: I-temporal migration, II-Spatial migration, III-Spatiotemporal migration.

Employing GLAD maize and soybean map^13^, we evaluated the sample generation accuracy for both soybeans and non-soybeans. GLAD maize and soybean map (https://glad.earthengine.app/view/china-crop-map) is a 2019 national maize and soybean map produced using field survey samples and binary random forest, in which the R^2^ between soybean mapping area and statistical yearbook area can reach 0.93. It is considered to be a reliable reference for accuracy validation. Using the actual samples from 2019 to 2021, we generated soybean and non-soybean samples for different regions in 2019 via three different methods. Subsequently, we calculated the sample generation accuracy for each region, as delineated in Fig. 9. Broadly speaking, with the exception of Liaoning and Jiangsu, the generation accuracy exceeds 80% for soybean samples and 95% for non-soybean samples, indicating the efficacy of the method used. Among the three-generation methods applied for soybean samples–temporal migration, spatial migration, and spatiotemporal migration–the average accuracies were 87.32%, 86.49%, and 83.44%, respectively. Temporal migration within the same region proved to be superior, followed by spatial migration. The least accuracy occurs in spatiotemporal migration. We postulated that minor annual variations in climatic factors, such as temperature and precipitation, contribute less to negative effects on sample migration compared to spatial heterogeneity due to regional differences. Among all provinces, Heilongjiang demonstrated the best results with an average accuracy of 92.72%. Inner Mongolia, Anhui, and Henan, three major soybean-producing provinces, also reached approximately 90%. In these provinces, soybeans are extensively cultivated, leading to relatively continuous and dense area, resulting in high accuracy. In contrast, Liaoning and Jiangsu, characterized by complex planting structures and fragmented soybean planting areas, had an accuracy below 80%. This lower accuracy can be attributed to these agricultural complexities and the adverse impact of rainfall on image quality. Conversely, the generation of non-soybean samples illustrated commendable accuracy and robustness across various regions. In summary, our sample generation method demonstrated exceptional proficiency across diverse regions, delivering commendable outcomes in both temporal and spatial sample generation.

### Soybean map and accuracy assessment

**Fig. 10:**
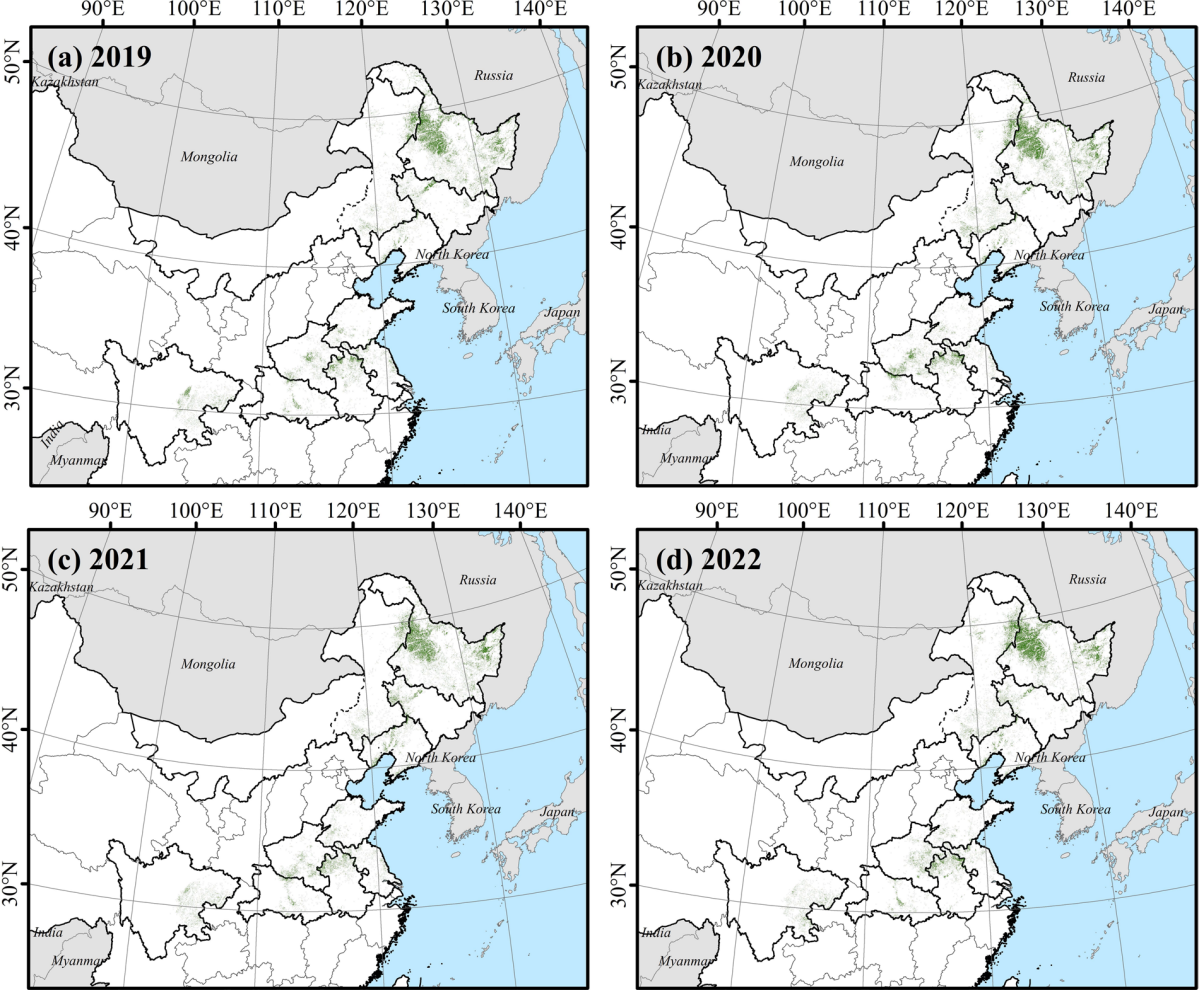
The crop maps in the main soybean producing area of China in (**a**) 2019, (**b**) 2020, (**c**) 2021, and (**d**) 2022.

By harnessing Sentinel-2 remote sensing imagery, selected Sentinel-1 SAR image data, ground surveys and generated samples, we mapped soybean planting areas for ten provinces nationwide (Fig. 10), denoted as ChinaSoybean10. We conducted accuracy assessments of the mapping results using both ground surveys and generated samples. The result indicates that in the northeastern region, the average overall accuracy for soybean planting areas mapping was 93.70%, with a prevailing Kappa coefficient of 0.8624. In crucial soybean cultivation areas of the Huang-Huai-Hai Plain, the middle-lower reaches of Yangtze River Plain, and Sichuan, the average overall mapping accuracy was 93.16%, accompanied by a Kappa coefficient of 0.7980 (Table 6). Moreover, we calculated both the producer’s accuracy and the user’s accuracy for each province. In the prominent soybean planting areas of the Northeast, the average producer’s accuracy, user’s accuracy, and F1-score were 92.23%, 88.70%, and 90.06%, respectively. For the Huang-Huai-Hai Plain, the Middle-Lower Yangtze Plain, and Sichuan, the average of these indicators were 80.15%, 89.59%, and 0.8434, respectively.

**Table 6:** Soybean Classification Mapping Overall Accuracy, Kappa, PA & UA.

Region	Year	OA	Kappa	PA	UA	F1
Northeast	2019*	0.9457	0.8937	0.9753	0.8539	0.9045
	2020*	0.9464	0.8733	0.9225	0.9047	0.9112
	2021*	0.9227	0.8493	0.8898	0.9175	0.9022
	2022*	0.9333	0.8337	0.9017	0.8718	0.8845
HHH-MLY	2019*	0.9525	0.8268	0.8474	0.8724	0.8561
	2020*	0.9361	0.7834	0.8123	0.8418	0.8227
	2021	0.9345	0.7580	0.7549	0.8434	0.7940
	2022	0.9452	0.7515	0.7632	0.8132	0.7835
Sichuan	2019	0.9399	0.8605	0.8468	0.9691	0.9038
	2020	0.9309	0.8384	0.8198	0.9681	0.8878
	2021	0.9219	0.8142	0.7748	0.9885	0.8687
	2022	0.8919	0.7512	0.7928	0.8713	0.8302

*Includes ground survey samples and generated samples, and the rest only have generated samples; HHH-MLY represents the Huang-Huai-Hai Plain and the Middle-Lower Yangtze Plain.

**Fig. 11:**
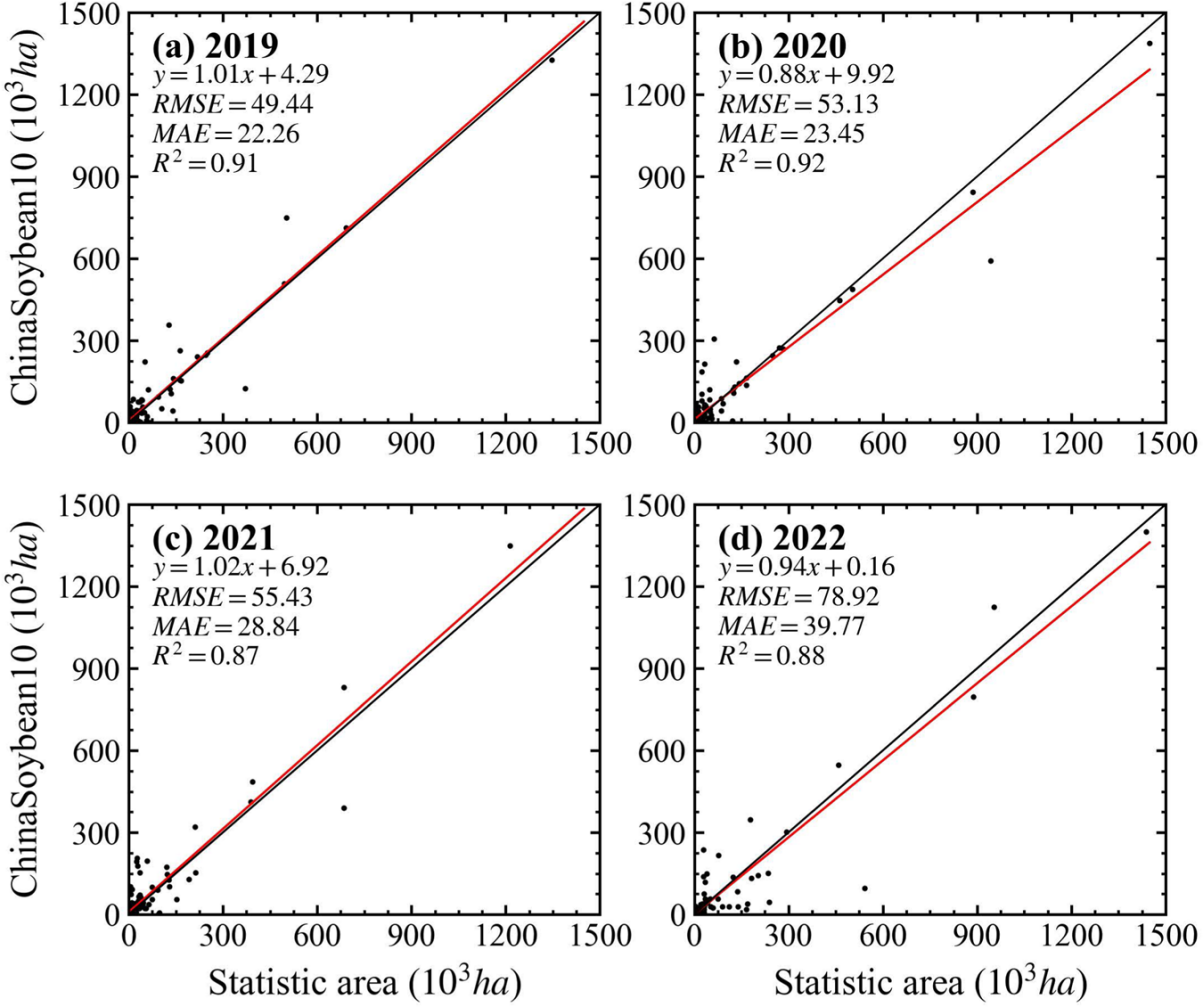
Comparison of mapped soybean area and planted soybean area reported by statistics at prefectural levels in (**a**) 2019, (**b**) 2020, (**c**) 2021, (**d**) 2022.

We compared the mapped soybean cropland areas of various prefecture-level cities with the officially reported planting areas, and quantitatively analyzed the accuracy of our soybean map by calculating R-squared (*R*^2^), root-mean-square-error (RMSE), and mean-absolute-error (MAE). The results demonstrate a high level of consistency between our annual soybean maps and official statistical data (*R*^2^ > 0.85), with values of 0.91, 0.92, 0.87, and 0.88 for the years 2019 to 2022, respectively (Fig. 11). *R*^2^ for 2019 and 2020 were both above 0.9, while there was a slight decrease in 2021 and 2022, likely due to the increased use of generated soybean samples in those years. In Fig. 11, we plotted the 1:1 line, and some prefecture-level cities with higher soybean production, such as Heihe, Qiqihar, and Hulunbuir, tended to cluster around this line. However, certain cities in the Huang-Huai-Hai and Yangtze River regions exhibited slight discrepancies compared to the statistical yearbook, possibly due to the more complex planting structures and the presence of numerous smallholders^65,66^, resulting in significant field-level heterogeneity. Nevertheless, our method consistently produces highly reliable estimates of planting area.

### Visual comparison with other products and methods

**Fig. 12:**
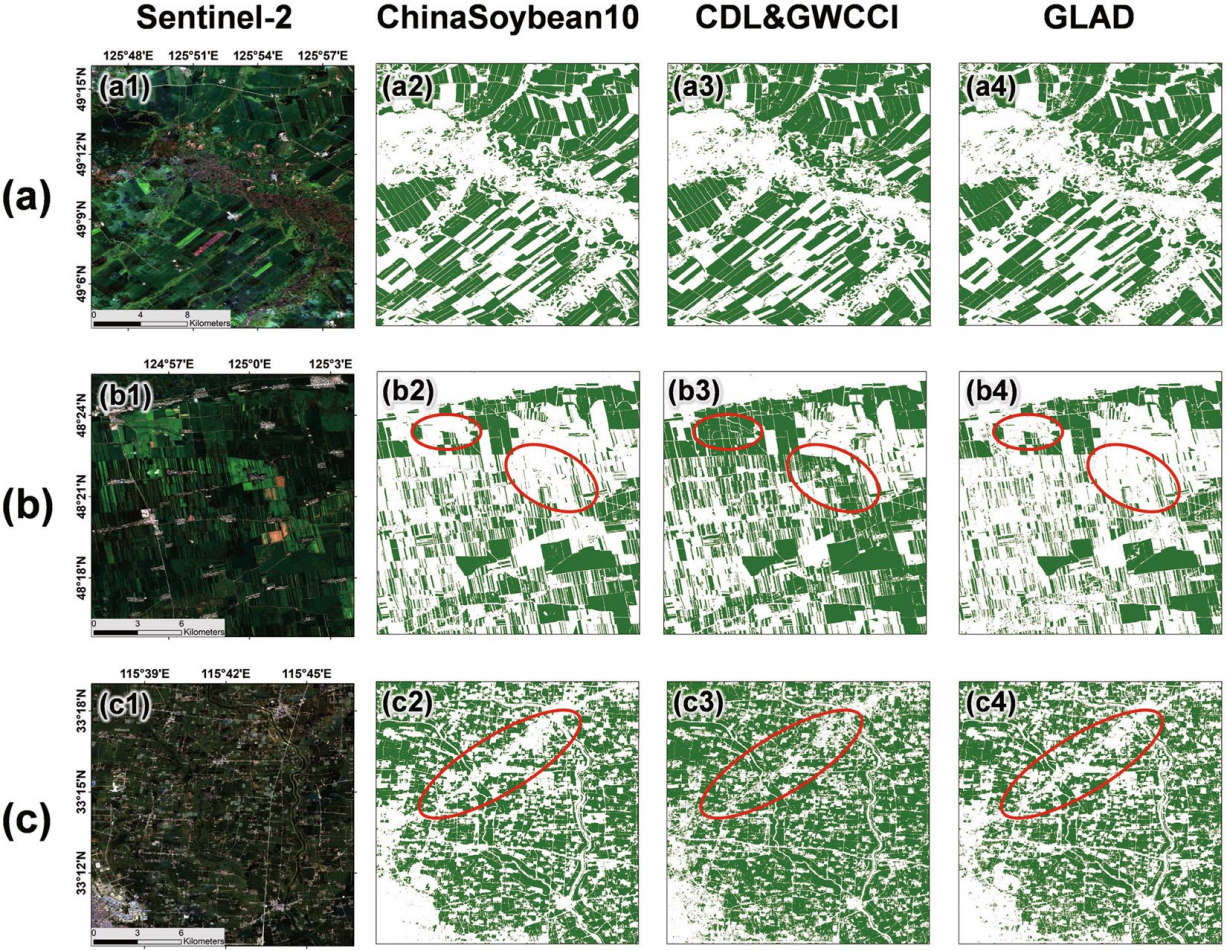
Comparison with Existing Methods and Results, the first column is a median-synthesized RGB image from Sentinel-2 after cloud removal (from DOY 200 to DOY 240); the second column represents ChinaSoybean10; The second column shows the results of CDL and the results extracted by GWCCI, where (c1-c2) are from CDL and (c3) is the extraction result of GWCCI.; the fourth column is the soybean map of GLAD, with (**a**–**c**) indicating different regions: (**a,b**) 2019 soybean map in Heilongjiang province; (**c**) 2019 soybean map in Anhui province.

Compared with some existing soybean distribution products, ChinaSoybean10 has wider spatial and temporal coverage. It also merges multi-source remote sensing data to achieve superior classification accuracy. We rank ChinaSoybean10 alongside GLAD maize-soybean map^13^, CDL (Crop Data Layer)^20^ and soybean map produced by GWCCI^27^. Among them, GLAD covers the soybean planting areas across the country in 2019, CDL covers the Northeast region, and GWCCI uses a common threshold of 0.17 for soybean mapping in all regions across the country. As a demonstration reference in 2019, we selected examples from Heilongjiang (Fig. 12a,b), and Anhui (Fig. 12c) to illustrate the comparison between our soybean mapping results and those of state-of-the-art methods. For the first example (Fig. 12a), our soybean mapping results are in good agreement with CDL and GLAD, reflecting the accuracy of our results. In the second example (Fig. 12b), our results are very similar to GLAD, while CDL has redundant soybean recognition results in the red-boxed area. The third example is in Anhui Province (Fig. 12c). CDL does not cover this area, so the GWCCI generated results are compared with our results. The soybean mapping effectiveness of GWCCI relies heavily on image quality and threshold selection. It can be found that the overall result contains more salt and pepper noise, as well as misclassified pixels in the red-boxed area (Fig. 12c3), which do not exist in our results and GLAD. The above comparison verifies the accuracy of our results.

### Advantages of the sample migration method

**Fig. 13:**
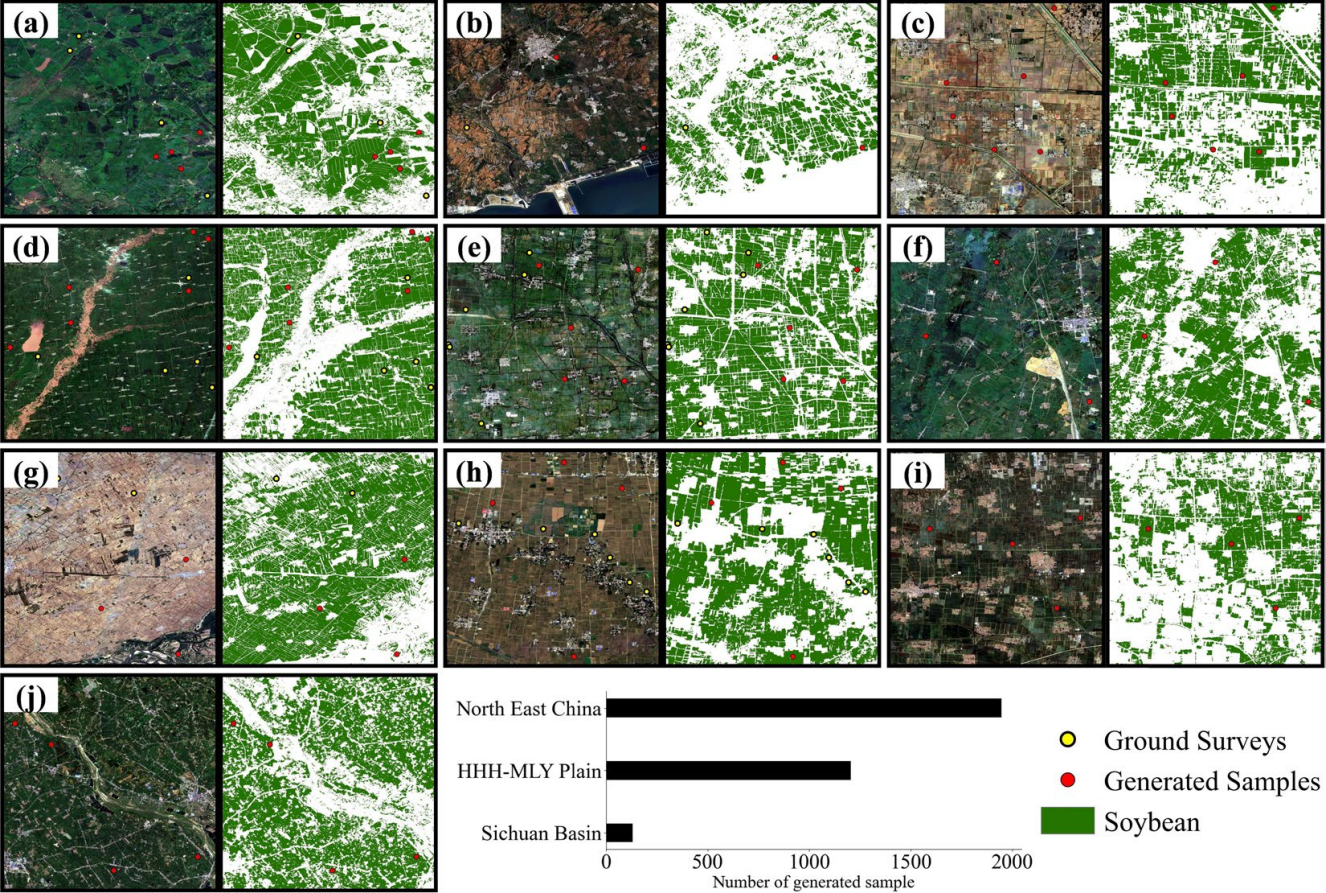
2019 generated soybean sample and ground surveys in 10 provinces. (**a**) Heilongjiang, (**b**) Inner Mongolia, (**c**) Jilin, (**d**) Liaoning, (**e**) Anhui, (**f**) Henan, (**g**) Shandong, (**h**) Hubei, (**i**) Jiangsu, (**j**) Sichuan.

The paper presents a soybean sample migration method, optimizing the acquisition of crop samples for soybean area mapping. The method is less financially demanding, automated, and provides accurate soybean and non-soybean samples using minimal pre-existing ones. Figure 13 displays samples taken in 10 provinces during 2019. The spatiotemporal migration is based on the disparity in crop’s band reflectivity and vegetation indexes. Using the integration of this curve during the growing season improves migration accuracy. The method’s potential lies in reducing the spatiotemporal heterogeneity of soybean phenology by using automatically determined growth season intervals.

Currently, there has been considerable research on no sample crop mapping based on crop knowledge and rule thresholds^25,27,32^. However, the effectiveness of these methods is severely hampered by threshold selection and are susceptible to clouds and fog, thus posing difficulties for large-scale crop mapping. The method presented in this paper selects random crop points based on the feature distribution of soybean samples, automatically determining filtering thresholds through the distribution characteristics of ground survey samples, thus enhancing its generality. Additionally, the mapping strategy in this paper combines generated samples with supervised classification. The thresholds mainly restrict the quality and quantity of samples, rather than directly affecting the mapping results. Therefore, threshold calculation parameters can be more “extreme” to obtain a purer set of soybean samples. Compared to the sample migration method based on DTW distance developed by Zhang *et al*.^33^, our method eliminates the need to obtain samples of other major crops in the target area, hence offering a more versatile and convenient solution. Some researchers use crop samples generated by existing products for mapping^13,56^. Although this method is convenient and efficient, it is subject to considerable limitations in terms of both time and space.

In conclusion, the soybean sample migration methodology elucidated in this paper adeptly and efficiently procures both soybean and non-soybean samples for regions devoid of such samples. This significantly aids in the creation of comprehensive crop mapping products and offers myriad possibilities for crop mapping.

## Usage Notes

China is the fourth largest soybean producer and the largest soybean importer in the world, and its soybean consumption relies heavily on imports. Mapping the distribution of soybean growing areas at the national scale is critical for food and energy security in the context of growing population and consumption. In this paper, we collected soybean field survey samples for many years and proposed a sample spatiotemporal migration method based on the temporal characteristics of vegetation index. Using field survey and generated samples, we create national 10 m soybean maps in China from 2019 to 2022. Through experiments, we found that the areas calculated by our soybean maps area consistent highly with the official statistical area at the prefecture-level. Therefore, our soybean mapping results can be used to support large-scale soybean yield estimates and quantitative analyzes of multi-year soybean-cultivated area changes. Furthermore, our datasets can serve as a reference and support uncertainty analysis for comparable products.

### Uncertainty

Despite our stringent data processing measures, certain sources of uncertainty remain inherent. Though we employed time-series Sentinel-2 data for soybean planting area mapping, the length of the soybean growing season is geographically variable. The 5-day revisit cycle might not consistently yield complete time-series spectral curves due to obstacles such as cloudy conditions and rainfall. While we successfully integrated Sentinel-1 data in certain regions and years, completely eradicating the speckle noise remains a complex task. Further, the widespread practice of soybean intercropping with crops such as corn and sorghum presents a substantial challenge in accurately mapping soybean’s spatial distribution within the Huang-Huai-Hai and Yangtze River regions. The potential mixed pixel effect arising from the 10-meter spatial resolution of Sentinel-2 data inevitably weakens the identification signal of specific crops, introducing uncertainty. Lastly, despite the validation of our sample generation method using ground-truth samples and existing products by achieving approximately 90% sample accuracy, potential deviations may still affect our results. Additionally, our methodology still relies upon terrestrial survey soybean samples. Ensuring minor phenological differences between these field survey samples, and generated samples is critical to the accuracy of sample generation. Looking forward, leveraging crop-specific maps, and highly resolved remote sensing products could offer solutions for the mixed pixel issue and enhance sample generation methods for optimal differentiation between soybean and non-soybean areas. Consequently, this could simplify the soybean extraction process.

## Data Availability

The programs used to generate the datasets and all the results were ESRI ArcGIS (10.6), Python (3.7 or 3.8) and Google Earth Engine (GEE). The scripts utilized for ChinaSoybean10 described in this paper can be accessed at https://github.com/ZihangLou/ChinaSoybean10.
