# Author Correction: Mapping annual 10-m soybean cropland with spatiotemporal sample migration

**DOI:** 10.1038/s41597-024-03430-w

**Published:** 2024-05-31

**Authors:** Hongchi Zhang, Zihang Lou, Dailiang Peng, Bing Zhang, Wang Luo, Jianxi Huang, Xiaoyang Zhang, Le Yu, Fumin Wang, Linsheng Huang, Guohua Liu, Shuang Gao, Jinkang Hu, Songlin Yang, Enhui Cheng

**Affiliations:** 1 Key Laboratory of Digital Earth Science, Aerospace Information Research Institute, Chinese Academy of Sciences, Beijing, 100094 China; 2 International Research Center of Big Data for Sustainable Development Goals, Beijing, 100094 China; 3 University of Chinese Academy of Sciences, Beijing, 100094 China; 4 Jiangxi Nuclearindustry Surveying and Mapping Institute Group Co., Ltd, Nanchang, 330038 China; 5 College of Land Science and Technology, China Agricultural University, Beijing, 100083 China; 6 Geospatial Sciences Center of Excellence, Department of Geography Geospatial Sciences, South Dakota State University, Brookings, SD 57007 USA; 7 Department of Earth System Science, Tsinghua University, Beijing, 100084 China; 8 Institute of Applied Remote Sensing & Information Technology, Zhejiang University, Hangzhou, 310058 China; 9 National Engineering Research Center for Agro-Ecological Big Data Analysis & Application, Anhui University, Hefei, 230601 China; 10 Innovation Academy for Microsatellites, Chinese Academy of Sciences, Shanghai, 200120 China

**Keywords:** Environmental sciences, Ecology, Environmental sciences, Ecology

Correction to: *Scientific Data* 10.1038/s41597-024-03273-5, published online 02 May 2024

In Fig. 11d of this article the Root Mean Square Error (RMSE) and Mean Absolute Error (MAE) were incorrectly given as 0.00 and 97.78 but should have been 78.92 and 39.77, respectively; the original and corrected Figure 11 are shown below. The original article has been updated.

The corrected version of Figure 11 is:

The original version of Figure 11 is: 

